# Two-step polyhydroxybutyrate production from hydrogenic effluent by freshwater microalgae *Coelastrella* sp. KKU-P1 and *Acutodesmus* sp. KKU-P2 under mixotrophic cultivation

**DOI:** 10.1016/j.heliyon.2024.e37261

**Published:** 2024-09-03

**Authors:** Kamolwan Thepsuthammarat, Tsuyoshi Imai, Pensri Plangklang, Sureewan Sittijunda, Alissara Reungsang

**Affiliations:** a Faculty of Environment and Resource Studies, Mahidol University, Nakhon Pathom, 73170, Thailand; b Division of Environmental Science and Engineering, Graduate School of Science and Engineering, Yamaguchi University, Yamaguchi, 755‐8611, Japan; c Department of Biotechnology, Faculty of Technology, Khon Kaen University, Khon Kaen, 40002, Thailand; d Research Centre for Sustainable Process Technology (CESPRO), Faculty of Engineering and Built Environment, University Kebangsaan, 43600, Malaysia; e Research Group for Development of Microbial Hydrogen Production Process from Biomass, Khon Kaen University, Khon Kaen, 40002, Thailand; f Academy of Science, Royal Society of Thailand, Bangkok, 10300, Thailand

**Keywords:** Zero-waste concept, Biodegradable polymers, Nutrient deficient, Bioplastic, Dark fermentative effluent

## Abstract

This study aimed to produce PHB using hydrogenic effluent discharged from the biohydrogen production process with freshwater microalgae including *Coelastrella* sp. KKU-P1, and *Acutodesmus* sp. KKU-P2. Batch experiments explored the influence of initial pH and hydrogenic effluent concentration, revealing optimal conditions at 10 % (v/v) effluent concentration and a pH of 6.5 for both KKU-P1 and KKU-P2. Subsequently, medium formulation and photoperiods were optimized to maximize biomass and PHB accumulation. The results showed that the optimal condition for PHB accumulation with KKU-P1 and KKU-P2 was nitrogen phosphorus (NP)-limited Bold's Basal Medium (BBM) under dark conditions. A two-step PHB accumulation in the upscale bioreactor was investigated under optimal conditions. The results showed that KKU-P1 achieved maximum PHB, protein, carbohydrate, and lipid contents of 4.57 %, 29.37 %, 24.76 %, and 13.21 %, respectively, whereas KKU-P2 achieved 6.35 %, 31.53 %, 16.16 %, and 4.77 %, respectively. Based on these findings, it appears that a mixotrophic approach under nutrient-limiting conditions is effective for PHB production in both KKU-P1 and KKU-P2 strains.

## Highlights

• Two-step PHB production from HE by freshwater microalgae was investigated.
• This is the first report on PHB production by microalgae using HE as the substrate.
• HE greater than 10 % (v/v) was found to reduce microalgal growth.
• Nutrient-limited mixotrophic approach is favorable for PHB production.
• PHB accumulation with KKU-P1 and KKU-P2 was optimal using NP-limited BBM under dark.

## Graphical abstract

**Figure:**
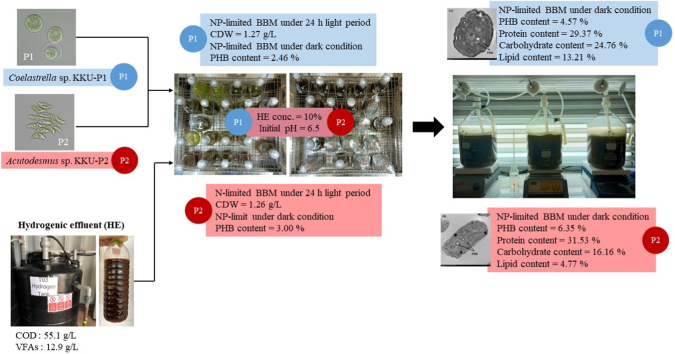

## 1 Introduction

One of the most pressing global environmental issues is the accumulation of petroleum-based plastic waste [1]. These plastics are not biodegradable and persist in the environment for extended periods, eventually breaking down into microplastics [1]. Moreover, the persistence of plastics and microplastics in the environment affects ecosystems through soil pollution from landfilling, marine and water pollution, and air pollution from open dumping. These problems accumulate throughout the entire life cycle of plastics and threaten ecological balance [2]. Therefore, the search for alternative eco-friendly polymers to replace petroleum-based plastics is an urgent and expanding field of interest [3]. Bioplastics, which are both eco-friendly and degradable by microorganisms, fall into four categories: photodegradable, semi-biodegradable, chemically synthesized, and polyhydroxyalkanoates (PHA) [4]. Among these, PHAs in particular have gained prominence as a novel class of bioplastics, and are further categorized into short-, medium-, and long-chain-length PHAs. PHA bioplastics are utilized in various consumer products, including films, absorbable structures, bone plates, and drug carriers [4]. Polyhydroxybutyrate (PHB), a type of short-chain-length PHA, is commonly found in a range of prokaryotic organisms. The material properties of PHB, such as its melting temperature and water resistance, are comparable to those of polypropylene [5,6]. In aquatic systems, plastics derived from PHB will sink and undergo degradation through biogeochemical processes, owing to the high density of PHB [5].

Several studies have reported that microorganisms, such as bacteria and microalgae, can intracellularly accumulate or produce PHB [[7], [8], [9]]. Photoautotrophic PHB production has been observed in various strains of cyanobacteria and a few strains of green microalgae [[7], [8], [9]]. The production of PHB by microalgae is appealing because they can utilize both organic compounds and inorganic carbon (CO_2_) for growth and production through photosynthetic and heterotrophic metabolism [10]. Moreover, the study by Phalanisong, Plangklang [11] found that the use of mixotrophic conditions is suitable for promoting microalgal growth and PHA production, rather than autotrophic conditions using fresh algal consortium. As previously stated, bacteria and microalgae can intracellularly accumulate or produce PHB [12,13]. Nonetheless, PHB accumulation by microalgae can reach satisfactory levels under optimized conditions, such as light period, temperature, pH, carbon source, and nutrient composition [14]. Additionally, PHB production has been improved by genetically engineered microbes that accumulate PHB and have been transformed with genes encoding PHB synthesis enzymes (3-ketothiolase, acetoacetyl-CoA reductase, and PHB synthase) [5,15]. A two-step cultivation approach has been explored, which involves optimal conditions for the growth stage followed by nutrient limitation for the biomolecule accumulation stage. This method has been previously reported in bacterial PHB production. For instance, a study by Ronďošová, Legerská [16] enhanced the growth of *Cupriavidus necator* DSM 545 in the first step and then promoted PHB accumulation. The results indicated that at a glucose concentration of 10.8 g/L, with ammonium sulfate and phosphate buffer concentrations of 0.95 g/L and 60.2 mmol/L, respectively, the maximum biomass concentration reached 4.54 g/L. Conversely, to optimize PHB accumulation, the optimal medium composition was 6.7 g/L glucose, 0.60 g/L ammonium sulfate, and 20 mmol/L phosphate buffer, resulting in a PHB yield of 49.1 % (w/w) of dry biomass. The major advantage of this cultivation method is the simultaneous increase in biomass concentration and high accumulation of target products. Therefore, the application of the two-stage cultivation approach to maximize algal growth and PHB accumulation is still limited. Hence, this study aims to maximize microalgal growth and simultaneously accumulate PHB.

Hydrogenic effluent (HE), also known as dark fermentative effluent, contains nutrients such as carbon, nitrogen (N), phosphorus (P), and volatile fatty acids (VFAs). These cannot be discharged directly without treatment and can be utilized to produce high-value-added products like PHB using bacteria or microalgae due to its cost-effectiveness [17]. Microalgae cultivation in HE for PHB accumulation offers advantages over bacteria, particularly in CO_2_ capture. Microalgae have the ability to perform biological fixation of CO_2_ from the atmosphere through photosynthesis, converting it into biomass. However, bacteria do not possess this capability [18]. For example, a study was conducted using palm oil mill effluent as the substrate to produce PHB using cyanobacteria including *Synechocystis* sp., *Nostoc* sp., and *Chroococcus* sp. The results showed that at the glycerol concentration of 670 mg/L, UV-C irradiation time of 70 min, and 20 mg/L of additional iron was the suitable condition to promote the highest PHB production using *Synechocystis* sp. as the inoculum [9]. Additionally, microalgae can yield other high-value products, including lipids, proteins, and antioxidants, which vary with the microalgae species. However, the unstable composition and presence of toxic substances in HE wastewater are concerns that must be addressed to achieve high product yields. Thus, cultivating microalgae in wastewater represents a sustainable approach for both wastewater treatment and PHB production.

Key factors affecting microalgal growth and biomass production include carbon source and concentration, nutrient availability, light intensity and periods, and pH [[10], [11], [12]]. Optimal carbon sources and mineral proportions enhance growth and production. Excessive nitrogen boosts growth and protein production, while nitrogen or phosphorus shortages cause carbohydrate or lipid accumulation but reduce amino acid and protein production, slowing growth [[19], [20], [21]]. The pH of the culture medium also plays a role in microalgal growth and biomass production. The ideal pH varies by microalgal species but typically ranges from 6.8 to 8.0 [22]. Light intensity and photoperiods influence photosynthesis, electron transfer, ATP synthesis, and biomass production. High light intensity and long photoperiods can cause stress, promoting storage of lipids and PHA, but also risking photo-inhibition and reduced biomass [23,24]. Controlling environmental factors is crucial for optimal microalgal growth and biomass production.

In the current study, HE from biohydrogen production using molasses served as the substrate for PHB production by the freshwater microalgae strains *Coelastrella* sp. KKU-P1 and *Acutodesmus* sp. KKU-P2. These two microalgal strains have the capability to produce PHA and biochemical compounds like lipids, proteins, and carbohydrates [10,11]. Initially, the effects of initial pH and HE concentration were conducted in the batch experiment. Subsequently, medium formulation and photoperiods were optimized to enhance biomass and PHB accumulation, considering the suitable pH and HE concentration. Finally, a two-step process for biomass and PHB accumulation was explored in an upscaled bioreactor under optimal conditions.

## 2 Materials and methods

### 2.1 Microalgal cultivation

Two microalgae strains, *Coelastrella* sp. KKU-P1 (GenBank Accession No. MW581273) and *Acutodesmus* sp. KKU-P2 (GenBank Accession No. MW555785), were provided by Dr. Pensri Plangklang for use as inoculum in PHB production. These strains were isolated from a freshwater fishpond in Nakhon Ratchasima, Thailand, and cultivated using 3N Bold's basal medium (3N BBM) [25]. The compositions of 3N BBM and other media are detailed in Table 1. For inoculum cultivation, a 5 L photobioreactor (PBR) with a working volume of 4.5 L was used. The 3N BBM was sterilized in an autoclave at 121 °C for 30 min. Air with 10 % (v/v) CO_2_ was supplied to the culture at a rate of 0.2 vvm through a filter. The inoculum was incubated at 30 ± 2 °C in a temperature-controlled room with continuous agitation (200 rpm) and under continuous illumination (6000 lux) for 14 days. The growth profiles of *Coelastrella* sp. KKU-P1 and *Acutodesmus* sp. KKU-P2 were analyzed. Samples were collected every 24 h to measure OD_680_, cell dry weight (CDW), and nitrate concentration.

**Table 1:** Chemical compositions of 3N BBM, N-, P-, and NP-limited BBM.

Stock No.	Chemicals	Concentration of stock solution (g/L)	Volume of stock solution (mL/L)			
			3N BBM	N-limited BBM	P-limited BBM	NP-limited BBM
1	NaNO_3_	25	30	None	30	None
2	CaCl_2_⋅2H_2_O	2.5	10	10	10	10
3	MgSO_4_⋅7H_2_O	7.5	10	10	10	10
4	KH_2_PO_4_	17.5	10	10	None	None
5	K_2_HPO_4_	7.5	10	10	None	None
6	NaCl	2.5	10	10	10	10
7	ZnSO_4_⋅7H_2_O	8.82	1	1	1	1
	Na_2_MoO_4_⋅2H_2_O	1.19				
	Co(NO_3_)_2_⋅6H_2_O	0.49				
	MnCl_2_⋅4H_2_O	1.44				
	CuSO_4_⋅5H_2_O	1.57				
8	H_3_BO_3_	11.42	1	1	1	1
9	EDTA⋅Na_2_	50	1	1	1	1
	KOH	31				
10	FeSO_4_⋅7H_2_O with 1 mL concentrated H_2_SO_4_	4.98	1	1	1	1
11	HE	optimal concentration (section 2.3)	none	added	added	added

On day 7, 1.5 L of the medium was removed to serve as the inoculum source, after which fresh sterilized 3N BBM medium was added to the PBR to restore the volume to 4.5 L. This cultivation process was repeated to propagate the microalgae. Subsequently, the 1.5 L of medium was allowed to settle by gravitational force. It was then centrifuged at 4000 rpm for 5 min to harvest the cells, which were washed twice with distilled water before being used as inoculum.

### 2.2 HE preparation

HE from biohydrogen production, which utilized molasses as feedstock at the bio-hythane pilot plant in Khon Kaen University Science Park, Khon Kaen, Thailand, served as the substrate for PHB production. This effluent was centrifuged at 8000 rpm for 15 min and then filtered through a 1.2-μm glass microfiber filter. The initial composition of HE, including trace elements and COD, was analyzed using standard methods as presented in Table 2 [26,27]. Acetic, butyric, lactic, and propionic acids were measured using high-performance liquid chromatography (HPLC) [28], while the total sugar content was determined using the phenol-sulfuric acid method [29]. Before use, the HE was stored at −4 °C. The chemical composition of the HE is detailed in Table 2.

**Table 2:** The chemical characteristics of HE.

Parameter	Value
Sodium chloride (%)	0.05
Nitrogen (%)	0.10
Phosphorus (%)	0.01
Potassium (%)	0.34
Calcium (mg/L)	412.86
Magnesium (mg/L)	276.66
Copper (mg/L)	5.77
Ferrous (mg/L)	2,036.09
Manganese (mg/L)	38.60
Zinc (mg/L)	12.89
Sodium (mg/L)	125.09
Sulfate (SO_4_^2−^) (mg/L)	43.88
Chemical Oxygen Demand (COD) (mg/L)	55,100
Total sugar (g/L)	8.37
Lactic acid (g/L)	6.40
Butyric acid (g/L)	3.19
Acetic acid (g/L)	3.10
Propionic acid (g/L)	0.23

### 2.3 Effect of initial pH and HE concentration on PHB production by *Coelastrella* sp. KKU-P1 and *Acutodesmus* sp. KKU-P2

Batch fermentation was performed in a 500-mL Erlenmeyer flask with a working volume of 250 mL. *Coelastrella* sp. KKU-P1 and *Acutodesmus* sp. KKU-P2 at the concentration of 0.15 g/L were added to culture medium. The culture medium contained varying concentrations of HE at 0, 3, 5, 7, and 10 % (v/v). The initial pH of the culture medium was adjusted to a range of 5.0–6.5 by adding either 1 M HCl or 2 M NaOH, respectively. The cultures were incubated at 30 ± 2 °C with a shaking speed of 120 rpm on an orbital shaker, under continuous illumination (6000 lux) for 10 days. Samples were collected every 5 day to analyze CDW and PHB content. The optimal HE concentration and initial pH were applied in subsequent experiments. All experiments were conducted in triplicate.

### 2.4 Optimization of nutrient composition and photoperiods for biomass production and PHB accumulation

An evaluation of the effects of nutrient composition and photoperiods on biomass production and PHB accumulation in microalgae were conducted using 500-mL flasks with a working volume of 250 mL. The nutrient compositions tested were 3N, nitrogen (N-), phosphorus (P-), and NP-limited BBM. N-, P-, and NP-limited BBM were supplemented with HE at the optimal concentration identified in Section 2.3 (Table 1). The presence of nitrogen and phosphorus in the HE backgrounds causes the medium to have a lower concentration of these elements compared to 3BBM. Therefore, the N-, P-, and NP-limited BBM still has a low concentration of nitrogen and phosphorus. The initial pH of the medium was adjusted to the optimal pH determined in Section 2.3. Photoperiods were set at 0, 12, and 24 h. The flasks were incubated at 30 ± 2 °C and agitated at a speed of 120 rpm on an orbital shaker with varying photoperiods for 10 days. Samples were collected every 5 day to determine CDW, pH, VFAs, chemical oxygen demand (COD), total N (TN), total phosphorus (TP), and PHB content. The optimal conditions identified in the flask experiments were applied to upscale cultivation in a 5 L PBR. All experiments were conducted in triplicate.

### 2.5 Two-step PHB production from HE using *Coelastrella* sp. KKU-P1 and *Acutodesmus* sp. KKU-P2 in an up-scale PBR

**Fig. 1:**
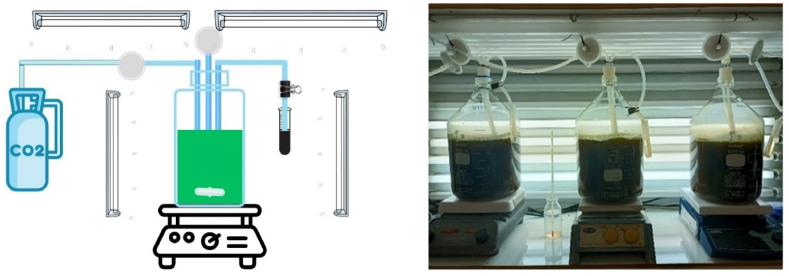
The schematic diagram and images of 5 L of photobioreactor.

Two-step PHB production from HE was conducted in a 5 L PBR with a working volume of 4.5 L and air containing 10 % CO_2_ (v/v) (Fig. 1). The PBR operated at 30 ± 2 °C in a temperature-controlled room with continuous agitation at 200 rpm. Initially, microalgal growth was maximized during the growth phase under optimal conditions identified in Section 2.4 for 5 days. At the end of this phase, microalgal cells were harvested to serve as inoculum for the subsequent PHB accumulation phase. The nutrient composition and photoperiods of this phase were adjusted according to the optimal PHB accumulation conditions detailed in Section 2.4. The PHB accumulation step involved a fermentation period of an additional 5 days. Throughout the cultivation, samples of fermentation media and microalgal cells were collected for pH, VFAs, COD, TN, TP, CDW, and PHB content and concentration analyses. On day 5, both microalgal cells were collected to assess protein, carbohydrate, and lipid contents. Additionally, the functional groups of the extracted PHB were characterized using attenuated total reflectance-Fourier transform infrared (ATR-FTIR) spectroscopy. Transmission electron microscopy (TEM) was employed to examine PHB accumulation within the microalgal cells. All experiments were conducted in triplicate.

### 2.6 Analytical and statisical methods

Culture samples were collected to determine the pH using a pH meter (Oakton, pH 700, USA). The optical density of the algal biomass was measured at 680 nm with a UV–VIS spectrophotometer (Shimadzu, UVmini-1240, Japan). The samples were then centrifuged at 10,000 rpm for 2 min to separate the cell pellets from the supernatant. The cell pellets were washed twice with distilled water and dried at 80 °C until a constant weight was achieved to measure the CDW. Intracellular PHB was extracted and measured by HPLC following the methods of Fernandes Júnior, Jansen [30]. Briefly, 3 mL of cell suspension were centrifuged, transferred to test tubes, and dried at 90 °C in hot air oven overnight. The cell contents were digested with 1 mL of 98 % sulfuric acids in a boiling water bath for 45 min. The digested suspension was then diluted with 4 mL of 4 mM sulfuric acid, mixed, and cooled on ice. It was subsequently filtrated using 0.45 μm of filter. The PHB concentration was determined using HPLC with an Aminex HPX-87H column, a UV detector, and 4 mM sulfuric acid as the mobile phase. For the TEM analysis, the microalgal cells were first fixed in 2.5 % (w/v) glutaraldehyde soluble in sodium phosphate buffer (pH 7.2) for 12 h at 4 °C, then washed with sodium phosphate buffer for 10 min (3 times) to remove any remaining glutaraldehyde. The cells were then fixed in 2 % osmium tetroxide for 2 h at room temperature, then washed using distilled water (three times for 10 min). The water inside the cells was removed using an acetone series at 30 %, 50 %, 70 %, 90 %, and 100 %, each step lasting 10 min, with the 100 % acetone step repeated three times. The cell contents were then replaced using mixture of acetone/plastic mixture at 2:1 1:1 and 1:2, each step performed for 3 h. Next, the pure plastic mixture was added three times, each step lasting 6 h. The cells were dried at 70 °C under a vacuum oven pump for 8 h. The microalgal cell were then cut into 70-nm sections using ultramicrotome (EM UC7, Leica, Austria), stained with 5 % uranyl acetate and lead citrate according to the method of Reynolds ES (1963), and imaged with TEM (Hitachi, HT7700, Japan) at an electric potential difference of 80 keV [31]. The functional groups of the extracted PHB were analyzed using an ATR-FTIR spectrometer (Agilent Technologies, USA). The extraction of PHB for FTIR analysis was described by Sitthikitpanya, Sittijunda [32]. Protein and carbohydrate contents in the microalgal cells were analyzed following the Lowry method [29]. Briefly, 0.5 mL of digested solution was added to a test tube containing 2.5 mL of reagent D (2.0 % Na_2_CO_3_ in NaOH, 0.5 % CuSO_4_·5H_2_O, and 1.0 % sodium potassium tartrate; all percentages are w/v). The mixture was then incubated for 10 min at room temperature. Subsequently, 0.25 mL of Folin-Ciocalteu was added, and the mixture was incubated at room temperature for 30 min. The protein concentration was then analyzed using a spectrophotometer at a wavelength of 660 nm. For carbohydrate analysis, 0.5 mL of digested solution was added to 0.5 mL of 5 % (w/v) phenol solution, followed by the addition of 2.5 mL sulfuric acid. The mixture was agitated for 10 s and then incubated in room temperature for 30 min. The carbohydrate content was analyzed using spectrophotometer at the wavelength of 490 nm [29]. To determine lipid content, 100 μL of the digested solution was added to 1 mL of sulfuric acid in a test tube. The mixture was heated for 10 min and subsequently cooled on ice for 5 min. The mixture was added to 2.5 mL of phosphovanillin reagent and agitated at 200 rpm at 37 °C for 15 min. The lipid content was then measured using a spectrophotometer at a wavelength of 530 nm [33].

The fermentation broth was collected to analyze nitrate concentration according to the method of the APHA. [27], TP using the HACH method 10127, TN using the HACH method 10072, and COD using the Merck COD cell tests method 114555 with a UV–VIS spectrophotometer (Merck, Pharo 300, Germany). The VFA concentration was determined using HPLC (Shimadzu, LC-20AD, Japan) as described by Sitthikitpanya, Reungsang [28]. The chemical characteristics of HE were analyzed using the method of APHA [27]. Total sugar concentration was examined using the phenol-sulfuric acid method described by Dubois, Gilles [34]. Both TS and VS were quantified using the APHA method.

The PHB content (%) was calculated by dividing the PHB production (mg/L) by the biomass concentration (mg/L) and multiplying by 100. The nitrate consumption (%) was calculated by subtracting the initial nitrate concentration from the final nitrate concentration and dividing it by the initial concentration, then multiplying by 100 %. Analysis of variance followed by Duncan's multiple range test and independent sample T-test were performed at a significance level of *p* < 0.05. SPSS version 25 software (SPSS, Inc., Chicago, IL, USA) was used for statistical analysis.

## 3 Results and discussion

### 3.1 The HE composition and growth profile of microalgae KKU-P1 and KKU-P2

The initial pH of HE was 5.0, with TS, VS, and total sugar concentrations of 35.00, 24.45, and 8.37 g/L, respectively. It also contains trace element COD values and VFAs, as detailed in Table 2. The predominant VFA in the HE was lactic acid at 6.40 g/L, followed by butyric, acetic, and propionic acids. These VFAs, being short-chain organic acids, serve as organic carbon sources for dinoflagellate growth [35]. Previous research indicated that *Acutodesmus* sp. KKU-P2 could utilize acetic acid from the effluent as a carbon source, thereby increasing biomass [32]. Su, Song [36] found that VFAs facilitated the rapid proliferation of *Chlorella pyrenoidosa* under heterotrophic conditions, with algae reaching a maximum biomass concentration of 0.14 g/L at the optimal VFA ratio. Conversely, *Scenedesmus quadricauda* achieved the highest lipid content of 29.54 % at the optimal VFA ratio under heterotrophic conditions.

**Fig. 2:**
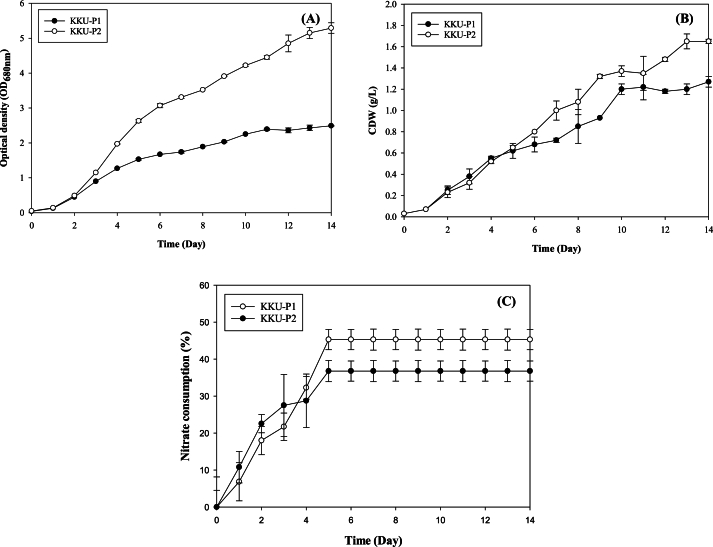
Growth profile of *Coelastrella* sp. KKU-P1 and *Acutodesmus* sp. KKU-P2 cultivated in 3N BBM medium for 14 days in terms of OD_680_ (A) and CDW (B) and changes of nitrate concentration (C). Mean values are average of three replications ± standard deviation (n = 3).

The growth profiles of *Coelastrella* sp. KKU-P1 and *Acutodesmus* sp. KKU-P2 were monitored by measuring OD_680_, CDW, and nitrate consumption, as depicted in Fig. 2. The results indicated that both KKU-P1 and KKU-P2 exhibited rapid growth, reaching peak levels by day 14 (Fig. 2). The growth profiles suggested that the minimal or absent lag phase for KKU-P1 and KKU-P2 could be due to CO_2_ supplementation [14]. KKU-P1 achieved a maximum OD_680_ of 2.49 and a CDW of 1.27 g/L (Fig. 2A and B). Similarly, KKU-P2 reached a maximum OD_680_ of 5.29 and a CDW of 1.65 g/L by day 14 (Fig. 2A and B), demonstrating a higher biomass yield than KKU-P1. Generally, biomass production in microalgae is associated with the N source. The nitrates consumption profiles revealed that the biomass of KKU-P1 and KKU-P2 increased sharply by day 5 and entered the stationary phase by days 10 and 13, respectively (Fig. 2C). Maximum nitrate consumption was recorded at 45.29 % for KKU-P1 and 36.76 % for KKU-P2 on day 5, suggesting that the cultivation process should optimally not exceed 10 days. These findings are consistent with those of Araujo, Silva [37], who reported that elevated sodium nitrate concentrations boosted the biomass production of *N. oculata* and *C. vulgaris*, but did not affect the growth of *T. chui*. High sodium nitrate consumption as a N source is crucial for microalgae survival.

### 3.2 Effect of initial pH and HE concentration on *Coelastrella* sp. KKU-P1 and *Acutodesmus* sp. KKU-P2 growth and PHB accumulation

**Fig. 3:**
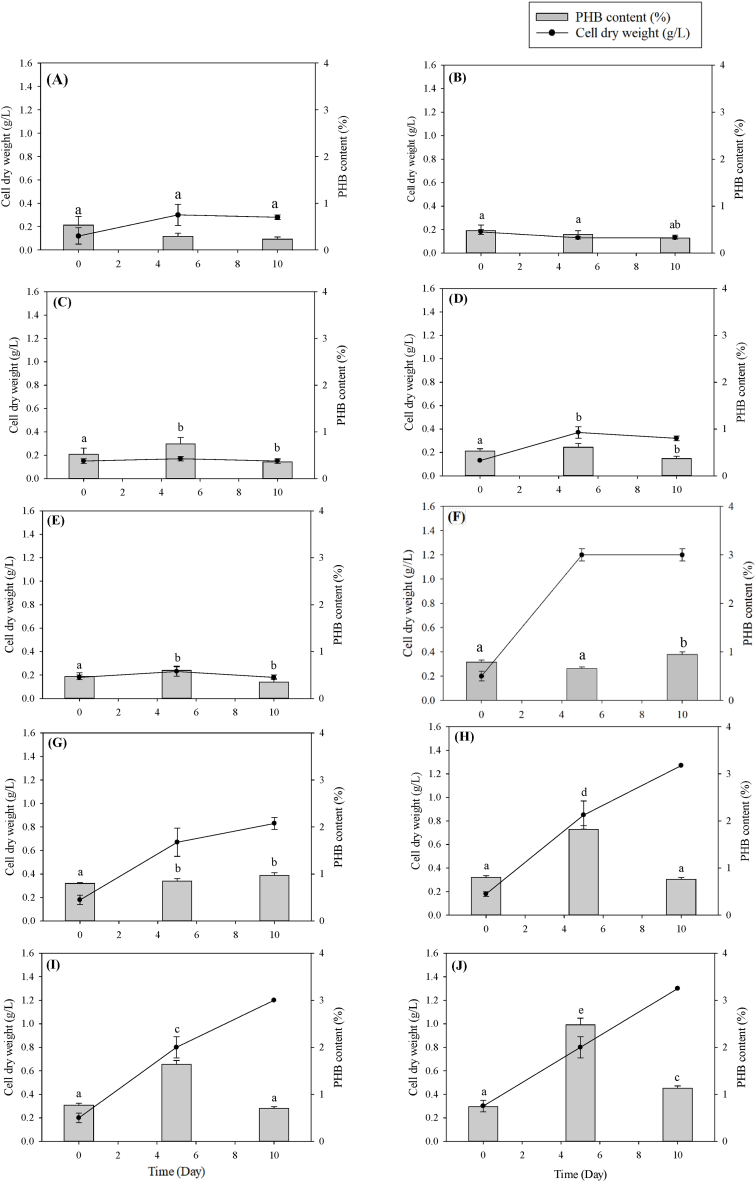
Time course profile of CDW and PHB content of *Coelastrella* sp. KKU-P1 using different HE concentrations and initial pH; group of pH 5: (A) 0 % HE, (B) 3 % HE, (C) 5 % HE, (D) 7 % HE, (E) 10 % HE; group of pH 6.5: (F) 0 % HE, (G) 3 % HE, (H) 5 % HE, (I) 7 % HE, and (J) 10 % HE. Mean values are the average of three replications ± standard deviation (n = 3). Statistical analysis was performed on the effects of pH and HE concentration on PHB content; values marked with the same letter are not significantly different (*p* < 0.05).

Fig. 3 illustrates the impacts of initial pH and HE concentration on the growth and PHB accumulation in *Coelastrella* sp. KKU-P1. The data indicated that variations in initial pH and HE concentration affected biomass and PHB production in KKU-P1. At an initial pH of 5.0 (Fig. 3A–E), the peak PHB content of 0.74 % was reached with a 5 % HE concentration (Fig. 3C), whereas the highest biomass production (0.37 g/L) was achieved with a 7 % HE concentration on day 5 (Fig. 3D) on the same day. Conversely, at an initial pH of 6.5, KKU-P1 attained a maximum biomass production of 1.30 g/L on day 10 and the highest PHB content of 2.48 % on day 5 with a 10 % effluent concentration (Fig. 3J). Notably, the PHB content on day 10 was lower than that on day 5 (Fig. 3J), a reduction likely due to PHB depolymerization, where PHB molecules decompose, yielding acetyl-CoA. This byproduct may then be utilized as an energy source or as precursor for synthesizing vital cellular components, as suggested by Singh, Sharma [6]. At pH 6.5, KKU-P1 exhibited a maximum PHB productivity of 0.150 mg/L·d at 10 % HE concentration on day 5, whereas at pH 5, the maximum PHB productivity of 0.041 mg/L·d was obtained at 5 % HE on day 5. Therefore, the highest PHB content and PHB productivity were achieved at pH 6.5. Interestingly, at pH 6.5, KKU-P1 took longer to achieve maximum biomass production than at pH 5. This extended period resulted in greater PHB accumulation compared to the shorter time frame at pH 5. These findings align with the study of Pezzolesi, Samorì [38], who found that slower growth rates often correlate with increased accumulation of storage compounds like PHB, as the microalgae redirect energy from growth to survival mechanisms.

**Fig. 4:**
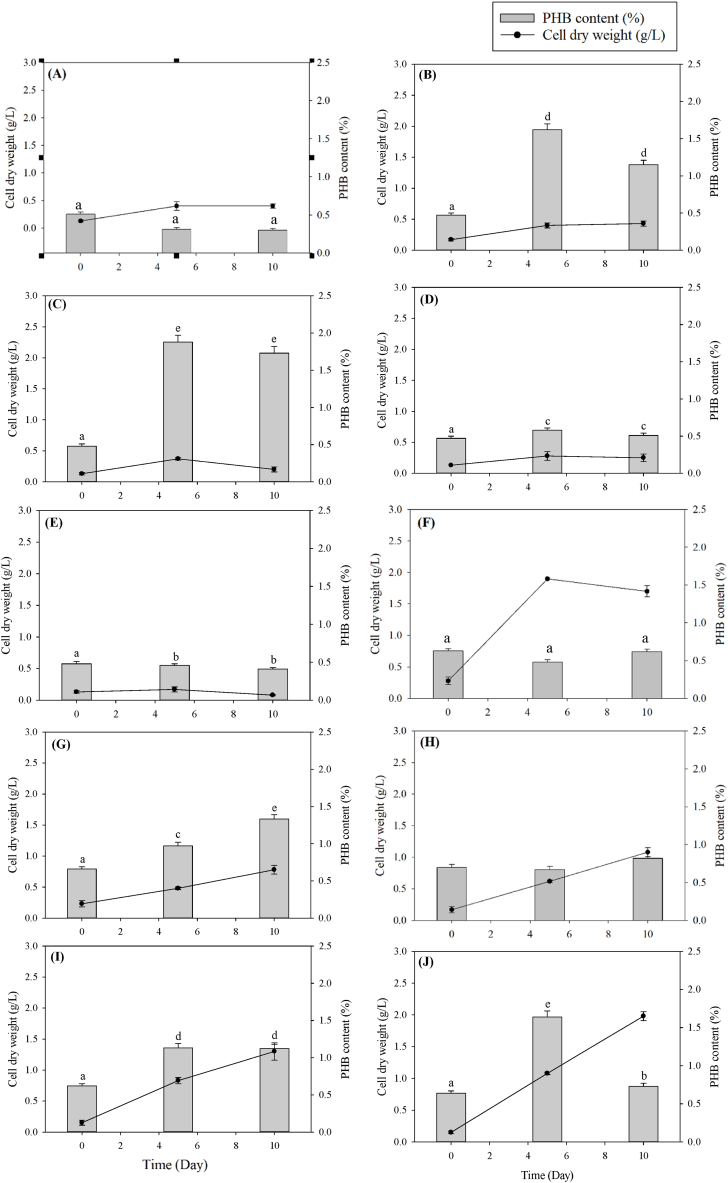
Time course profile of CDW and PHB content of *Acutodesmus* sp. KKU-P2 using different HE concentrations and initial pH; group of pH 5: (A) 0 % HE, (B) 3 % HE, (C) 5 % HE, (D) 7 % HE, (E) 10 % HE; group of pH 6.5: (F) 0 % HE, (G) 3 % HE, (H) 5 % HE, (I) 7 % HE, and (J) 10 % HE. Mean values are average of three replications ± standard deviation (n = 3). Statistical analysis was performed on the effects of pH and HE concentration on PHB content; values marked with the same letter are not significantly different (*p* < 0.05).

For *Acutodesmus* sp. KKU-P2, with an initial pH of 5 (Fig. 4A–E), the highest biomass production reached 0.43 g-CDW/L on day 10 at an HE concentration of 3 % (Fig. 4B). Conversely, the maximum PHB content and PHB productivity of 1.88 % and 0.670 mg/L·d were achieved on day 5 with a 5 % HE concentration (Fig. 4C). At an initial pH of 6.5 (Fig. 4F–J), increasing the HE concentration similarly elevated the PHB content as observed at pH 5. The peak biomass production of 1.98 g-CDW/L occurred on day 10, while the highest PHB content and PHB productivity of 1.64 % and 0.165 mg/L·d were recorded on day 5, both under conditions of 10 % HE supplementation at a pH of 6.5 (Fig. 4J).

The carbon source and its concentration are critical factors influencing microalgal growth and PHB production. Microalgae can grow photoautotrophically under low CO_2_ concentrations in the air, utilizing sunlight [39]. However, the addition of organic acids can enhance growth and product accumulation via heterotrophic metabolism. Al Battashi, Al-Kindi [40] observed that supplementary VFAs can boost PHB production, as organic carbon sources increase the Acetyl-CoA and NADH pool, essential precursors for PHB synthesis [14]. Furthermore, *Schizochytrium limacinum* showed promising biomass productivity when consuming acetic and butyric acids, and the use of VFAs derived from industrial waste as a carbon source resulted in biomass yields of 18.5 g/L, with 54.0 % lipids and 46.3 % docosahexaenoic acid (DHA) [41]. Amadu, Qiu [17] found that VFAs from the acidogenic fermentation of cassava starch manufacturing waste were effective substrates for PHB production. Additionally, waste substrates like anaerobic digester effluent, rich in VFAs, could serve as substrates for microalgal PHB production.

In conclusion, the optimal conditions for biomass production and PHB accumulation in KKU-P1 and KKU-P2 were identified as an initial pH value of 6.5 and a HE concentration of 10 %. These findings are consistent with those of Ratnaningrum, Endah [42], who reported that an initial pH range of 6.0–7.5 is conducive to microbial growth and PHB production. Consequently, this optimal pH and HE concentration parameters were employed in subsequent experiments.

### 3.3 Optimization of nutrient composition and photoperiods for biomass production and PHB accumulation using *Coelastrella* sp. KKU-P1 and *Acutodesmus* sp. KKU-P2

**Fig. 5:**
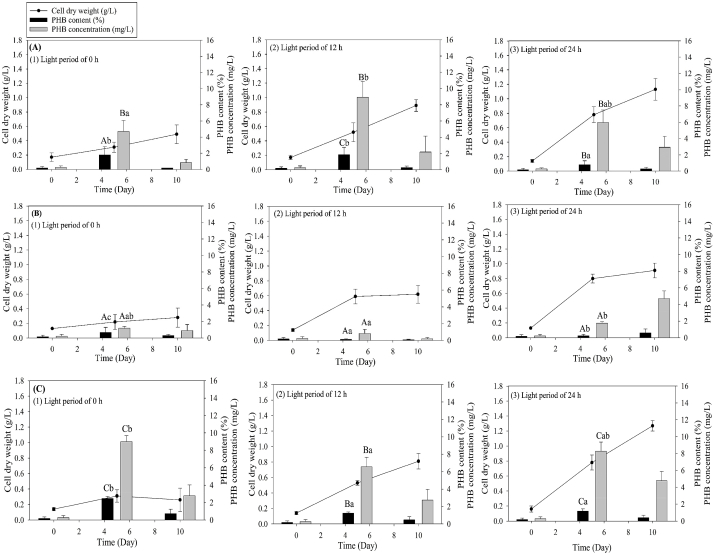
Change of CDW and PHB of *Coelastrella* sp. KKU-P1 cultivated in N-limited BBM (A), P-limited BBM (B), NP-limited BBM (C) with different photoperiods of 0 (1), 12 (2), and 24 (3) h. Mean values are the average of three replications ± standard deviation (n = 3). Statistical analysis was performed using the PHB content and concentration. Values marked with the same letter are not significantly different (*p* < 0.05). Uppercase letters compare the same photoperiod across different media formulations, while lowercase letters compare different photoperiods within the same medium formulation.

**Fig. 6:**
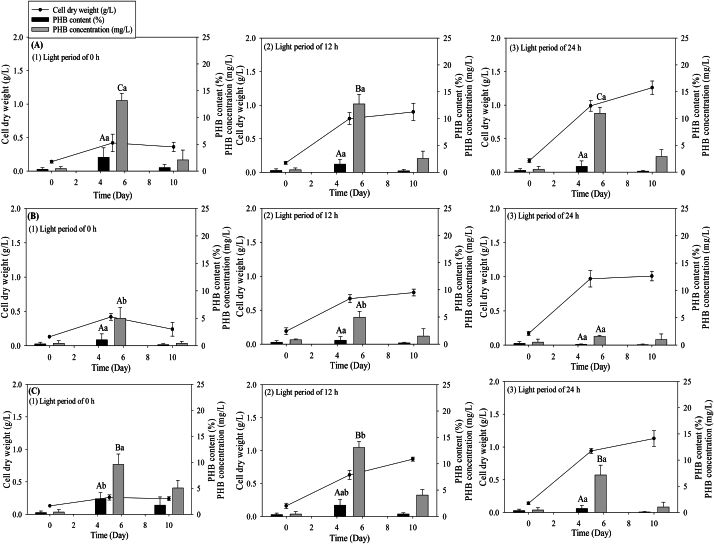
Change of CDW and PHB of *Acutodesmus* sp. KKU-P2 cultivated in N-limited BBM (A), P-limited BBM (B), and NP-limited BBM (C) with different photoperiods of 0, 12, and 24 h. Mean values are average of three replications ± standard deviation (n = 3). Statistical analysis was performed using the PHB content and concentration. Values marked with the same letter are not significantly different (*p* < 0.05). Uppercase letters compare the same photoperiod across different media formulations, while lowercase letters compare different photoperiods within the same medium formulation.

Fig. 5, Fig. 6 depict the effects of nutrient composition and photoperiods on biomass production and PHB accumulation in *Coelastrella* sp. KKU-P1 and *Acutodesmus* sp. KKU-P2. The cultivation of KKU-P1 and KKU-P2 under varying nutrient compositions and photoperiods resulted in altered biomass and PHB accumulation. Fig. 5 shows that KKU-P1 cultivated in N-, P-, and NP-limited BBM under continuous 24-h illumination achieved the highest biomass concentrations of 0.94, 1.13, 0.91, and 1.27 g-CDW/L, respectively. Conversely, the highest PHB content was observed with NP-limited conditions in the dark (0 h of light). Under these conditions, a PHB content of 2.46 % was recorded on day 5 (Fig. 5C(1)). However, extending the cultivation period from 5 to 10 d led to a reduction in PHB content due to PHB depolymerization, where PHB molecules break down into acetyl-CoA. This breakdown may serve as an energy source or as precursors for synthesizing essential cell components, as Singh et al. (2016) suggested.

Table 3 displays the substrate consumption and nutrient assimilation in terms of VFAs, COD, TN, and TP. The results indicate that KKU-P1 can utilize VFAs present in HE to support growth and PHB production. It also assimilates N and P, leading to reduced final concentrations of VFAs, COD, TN, and TP. The VFAs concentration decreased to zero, suggesting that KKU-P1 consumes all types of acids in HE, including acetic, butyric, and lactic acids. A previous report demonstrated that supplementing acetate as an organic carbon source for cultivating *Pectinodesmus* sp. increased lipid accumulation, which correlates with PHB accumulation [43]. Concurrently, the pH increased due to photosynthetic activity (Table 3). Microalgal photosynthesis involves CO_2_ fixation, which causes the separation of carbonate and bicarbonate and the release of hydroxyl ions into the culture medium, resulting in a pH increase [44]. Under NP-limited conditions without light (0 h), which is suitable for PHB accumulation, the removal rates of COD, N, and P were 70.08 %, 54.28 %, and 100 %, respectively. This indicates that PHB accumulation by KKU-P1 is closely related to P deficiency. These findings are consistent with the study by Samadhiya, Ghosh [43], which found that low P levels in the medium increased lipid accumulation. As mentioned, NP-limited BBM under a 24-h light period was the optimal condition for maximizing KKU-P1 growth during the first step growth phase, while NP-limited BBM in dark conditions was suitable for PHB production during the second step PHB accumulation phase.

**Table 3:** Change of parameters during cultivation of *Coelastrella* sp. KKU-P1 with different nutrient composition and light period.

Media	Light period	VFAs (g/L)		pH		COD (g/L)		TN (mg/L)		TP (mg/L)	
		Initial	Final	Initial	Final	Initial	Final	Initial	Final	Initial	Final
N-limited BBM	0 h	1.27 ± 0.02	0 ± 0.00^Aa^	6.51 ± 0.01	8.31 ± 0.28^Aa^	5.05 ± 0.09	1.44 ± 0.11^Aa^	96.54 ± 5.09	41.39 ± 2.15^Aa^	64.86 ± 3.01	52.70 ± 2.07^Bc^
	12 h	1.25 ± 0.06	0 ± 0.00 ^Aa^	6.51 ± 0.01	9.30 ± 0.92^Aab^	5.13 ± 0.21	1.36 ± 0.15^Aa^	91.23 ± 3.46	44.11 ± 6.30^Aa^	61.47 ± 4.20	43.73 ± 2.60^Bb^
	24 h	1.23 ± 0.07	0 ± 0.00 ^Aa^	6.51 ± 0.01	9.96 ± 0.65^Ab^	5.12 ± 0.06	1.44 ± 0.09^Aa^	98.54 ± 7.35	43.26 ± 4.56^Aa^	67.18 ± 4.17	36.43 ± 1.50^Ca^
P-limited BBM	0 h	1.28 ± 0.04	0 ± 0.00 ^Aa^	6.50 ± 0.01	8.50 ± 0.21^Aa^	5.09 ± 0.14	1.83 ± 0.35^Aa^	192.39 ± 3.07	84.63 ± 16.20^Ba^	21.45 ± 2.15	0 ± 0.00^Aa^
	12 h	1.20 ± 0.02	0 ± 0.00 ^Aa^	6.50 ± 0.01	10.40 ± 0.65^Ab^	5.06 ± 0.06	1.72 ± 0.09^Ba^	195.78 ± 5.14	71.02 ± 10.03^Ba^	24.45 ± 1.98	2.22 ± 1.14^Aa^
	24 h	1.27 ± 0.04	0 ± 0.00 ^Aa^	6.51 ± 0.01	10.52 ± 0.65^Ab^	5.14 ± 0.13	1.54 ± 0.11^Aa^	189.45 ± 2.48	68.82 ± 3.54^Ba^	22.45 ± 1.75	5.69 ± 2.07^Bb^
NP-limited BBM	0 h	1.21 ± 0.04	0 ± 0.00 ^Aa^	6.51 ± 0.01	8.23 ± 0.30 ^Aa^	5.18 ± 0.06	1.55 ± 0.28^Aa^	93.16 ± 3.68	42.59 ± 0.85^Aa^	19.79 ± 1.69	0 ± 0.00 ^Aa^
	12 h	1.18 ± 0.03	0 ± 0.00 ^Aa^	6.51 ± 0.01	9.21 ± 0.61^Aab^	5.15 ± 0.10	1.46 ± 0.23^Aba^	92.48 ± 4.85	43.35 ± 2.54^Aa^	20.32 ± 2.01	0 ± 0.00 ^Aa^
	24 h	1.30 ± 0.01	0 ± 0.00 ^Aa^	6.50 ± 0.01	9.79 ± 0.61 ^Ab^	5.12 ± 0.04	1.53 ± 0.45^Aa^	96.74 ± 5.72	40.09 ± 2.01^Aa^	18.97 ± 1.76	0.41 ± 0.21^Ab^

Mean values are the average of three replications ± standard deviation (n = 3). Values marked with the same letter are not significantly different (*p* < 0.05). Uppercase letters compare the same photoperiod across different media formulations, while lowercase letters compare different photoperiods within the same medium formulation.

For *Acutodesmus* sp. KKU-P2, the maximum biomass concentration (1.26 g/L) was achieved using N-limited BBM as the medium with continuous illumination (Fig. 6A(3)). Conversely, NP-limited BBM under dark conditions yielded the highest PHB content of 3.00 % on day 5 of cultivation (Fig. 6C(1)). The results indicated that reduced light exposure periods led to increased PHB accumulation. The substrate consumption and nutrient assimilation trends using KKU-P2 as the inoculum were analogous to those observed with KKU-P1. The VFAs, COD, TN, and TP decreased by the end of fermentation, while the pH increased (Table 4). This suggests that KKU-P2 could utilize the VFAs in the HE for growth and PHB accumulation. Moreover, under nitrogen and phosphorus limitation in the medium, KKU-P2 tended to accumulate PHB. Therefore, N-limited BBM with a 24-h light period was the optimal condition for maximizing KKU-P2 growth (first step growth phase), and NP-limited BBM under dark conditions were suitable for PHB production (second step PHB accumulation phase).

**Table 4:** Change of parameters during cultivation of *Acutodesmus* sp. KKU-P2 with different nutrient composition and light period.

Media	Light period	VFAs (g/L)		pH		COD (g/L)		TN (mg/L)		TP (mg/L)	
		Initial	Final	Initial	Final	Initial	Final	Initial	Final	Initial	Final
N-limited BBM	0 h	1.15 ± 0.05	0 ± 0.00^Aa^	6.51 ± 0.01	8.37 ± 0.31^Aa^	5.16 ± 0.09	1.33 ± 0.12^Aa^	100.54 ± 2.71	50.92 ± 2.00^Ab^	64.79 ± 2.11	51.75 ± 1.43^Ba^
	12 h	1.08 ± 0.10	0 ± 0.00^Aa^	6.50 ± 0.01	9.82 ± 0.36^Ab^	5.23 ± 0.13	1.31 ± 0.17^Aa^	102.83 ± 1.75	28.30 ± 1.05^ABa^	65.41 ± 3.54	28.78 ± 2.02^Ca^
	24 h	1.08 ± 0.10	0 ± 0.00^Aa^	6.50 ± 0.01	10.61 ± 0.51^Ab^	5.12 ± 0.07	1.45 ± 0.09^Aa^	98.87 ± 3.46	27.01 ± 1.31^Aa^	67.07 ± 4.03	23.13 ± 1.96^Ca^
P-limited BBM	0 h	1.04 ± 0.06	0 ± 0.00^Aa^	6.50 ± 0.01	8.55 ± 0.25^Aa^	5.16 ± 0.14	1.34 ± 0.08^Aa^	193.19 ± 2.40	66.90 ± 3.24^Bb^	21.30 ± 1.09	1.32 ± 0.21^Aa^
	12 h	1.08 ± 0.08	0 ± 0.00^Aa^	6.51 ± 0.01	10.48 ± 0.76^Ab^	5.13 ± 0.10	1.34 ± 0.11^Aa^	195.42 ± 1.87	31.18 ± 0.75^Aa^	20.85 ± 1.73	7.07 ± 0.26^Bc^
	24 h	1.10 ± 0.10	0 ± 0.00^Aa^	6.51 ± 0.01	10.56 ± 0.70^Ab^	5.11 ± 0.18	1.41 ± 0.13^Aa^	192.86 ± 2.31	66.56 ± 2.27^Cb^	21.77 ± 1.29	5.67 ± 0.71^Bb^
NP-limited BBM	0 h	1.19 ± 0.04	0 ± 0.00^Aa^	6.50 ± 0.01	8.15 ± 0.15^Aa^	5.10 ± 0.13	1.65 ± 0.24^Aa^	94.39 ± 0.75	48.61 ± 2.70^Aab^	21.29 ± 1.55	1.54 ± 0.11^Ab^
	12 h	1.09 ± 0.04	0 ± 0.00^Aa^	6.51 ± 0.01	9.65 ± 0.45^Ab^	5.06 ± 0.11	1.29 ± 0.22^Aa^	97.66 ± 1.12	45.20 ± 3.83^Ba^	20.58 ± 1.64	0.71 ± 0.19^Aa^
	24 h	1.17 ± 0.10	0 ± 0.00^Aa^	6.50 ± 0.01	10.65 ± 0.59^Ac^	5.13 ± 0.13	1.40 ± 0.15^Aa^	95.03 ± 0.96	54.37 ± 4.17^Bb^	20.72 ± 2.08	1.92 ± 0.20^Ac^

Mean values are the average of three replications ± standard deviation (n = 3). Values marked with the same letter are not significantly different (*p* < 0.05). Uppercase letters compare the same photoperiod across different media formulations, while lowercase letters compare different photoperiods within the same medium formulation.

Our findings indicate that the NP-limitation medium is suitable for promoting PHB accumulation in *Coelastrella* sp. KKU-P1 and *Acutodesmus* sp. KKU-P2. However, previous research has shown that PHB accumulation under P-limited conditions exceeds N deficiency [14]. Utilizing a P-limiting nutrient can enhance PHB production by elevating NADPH concentration and inhibiting citrate synthase activity, thereby increasing the availability of acetyl-CoA for PHB synthesis [19].

As mentioned, photoperiods affect microalgal growth and PHB accumulation. Under continuous illumination, KKU-P1 and KKU-P2 utilize light as an energy source and organic acids in HE as a carbon source for growth through photosynthetic and heterotrophic metabolism, resulting in maximum biomass. Conversely, PHB accumulation occurs in the absence of light. It can be concluded that reduced light exposure times lead to PHB accumulation, suggesting potential cost reductions. This finding aligns with the study by Cassuriaga et al. [29], which investigated PHB production from *Chlorella fusca* LEB 111 under varying photoperiods of 18, 16, and 6 h. They found that *C. fusca* LEB 111 could accumulate PHB during shorter photoperiods (6 h), with a maximum PHB production of 17.4 % observed. The alteration in the biochemical composition of *Chlorella* biomass may be caused by shorter photoperiods, resulting in changes in membrane permeability that affect nutrient assimilation and may induce PHB accumulation [45].

Furthermore, N is crucial for microalgal growth. Microalgae assimilate nutrients such as N and P during cultivation, incorporating them into their biomass and yielding a nutritionally rich product suitable for use as a food supplement. The composition of micro- and macronutrients influences the macromolecular content, including proteins, carbohydrates, and lipids. Typically, the variation in macromolecular content within microalgal cells is contingent upon nutrient availability. Under N-limited conditions, microalgal growth decreases, while lipid production increases [46]. Braga, Moreira [47] observed that microalgae alter their metabolic pathways to produce carbohydrates and/or lipids under N scarcity, leading to the accumulation of both. Carbohydrates are generally produced during CO_2_ fixation via photosynthesis. Conversely, PHB, categorized as a lipid, is synthesized from acetyl-CoA, which is a precursor for PHB synthesis. When nutrients are abundant, free CoA levels are high due to the demand for acetyl groups in carbon skeleton production and energy generation via the Krebs cycle. However, nutrient limitations reduce the need for acetyl groups, decreasing free CoA levels and, consequently, relieving the inhibition on β-ketothiolase, which initiates PHB synthesis [44]. P is also vital for synthesizing cellular components such as phospholipids, DNA, RNA, and ATP [46].

### 3.4 Two-step PHB production from HE using *Coelastrella* sp. KKU-P1 and *Acutodesmus* sp. KKU-P2 in an up-scale PBR

**Fig. 7:**
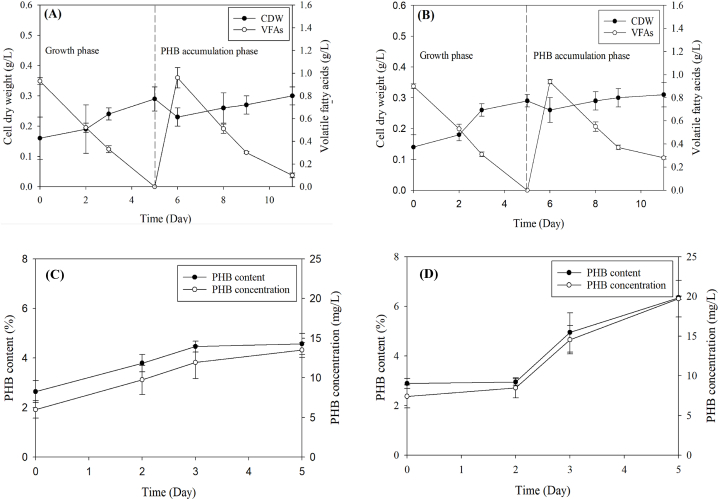
Change of CDW and VFAs during growth and PHB accumulation phase of *Coelastrella* sp. KKU-P1 (A) and *Acutodesmus* sp. KKU-P2 (B) and the PHB content and PHB concentration during the PHB accumulation phase of *Coelastrella* sp. KKU-P1 (C) and *Acutodesmus* sp. KKU-P2 (D). Mean values are average of three replications ± standard deviation (n = 3).

Two-step PHB production using *Coelastrella* sp. KKU-P1 and *Acutodesmus* sp. KKU-P2 is depicted in Fig. 7. The processes began with the enhancement of microalgal biomass concentration (growth phase), followed by inducing the microalgae to accumulate PHB (PHB accumulation phase). The results indicated that under 24-h photoperiods with NP-limited BBM, KKU-P1 exhibited rapid growth, attaining a peak biomass concentration of 0.29 g-CDW/L by day 5. After 5 day of cultivation, the medium was switched to NP-limited BBM and the conditions were changed to darkness (PHB accumulation phase). During this phase, the growth of KKU-P1 slightly decreased to 0.23 g-CDW/L before marginally rising to 0.30 g-CDW/L, corresponding with the reduction of VFAs from 0.96 to 0.10 g/L (Fig. 7A). The biomass concentration of KKU-P1 during the PHB accumulation phase was comparable to that of the growth phase. This suggests that KKU-P1 can utilize organic nutrients in HE and CO_2_ to optimize growth in the growth phase and then convert organic nutrients and CO_2_ into PHB during the accumulation phase. These findings demonstrate that KKU-P1 can use VFAs and CO_2_ as carbon sources for growth through heterotrophic and photosynthetic metabolism. In the PHB accumulation phase, KKU-P1 was able to accumulate PHB under nutrient-limited conditions, with the PHB content increasing from 2.64 % to 4.57 %, and the PHB concentration rising from 6.00 to 13.52 mg/L (Fig. 7C).

For KKU-P2, the biomass trend increased from day 0–5, reaching a maximum concentration of 0.29 g-CDW/L (Fig. 7B). This growth correlated with a decrease in VFA concentration by the end of day 5. In the PHB accumulation phase, the biomass concentration rose slightly to 0.31 g-CDW/L, while the VFA concentration decreased from 0.94 to 0.28 g/L. During this phase, a PHB content of 6.35 % and a PHB concentration of 19.72 mg/L were achieved (Fig. 7D). The slight increase in biomass during the PHB accumulation phase correlated with low nutrient consumption, such as COD, TN, and TP (Table 5). This indicated that KKU-P2 could utilize organic nutrients in the HE and CO_2_ for growth in the growth phase, where the highest biomass was observed. Price et al. [22] reported that cyanobacteria can produce PHB under photoautotrophic and heterotrophic conditions. However, certain cyanobacterial species can produce larger quantities of PHB under heterotrophic conditions. Supplementing with CO_2_ may enhance the growth and PHB accumulation in microbes, considering the low CO_2_ concentration in ambient air. Furthermore, nutrient-rich conditions could also promote PHB accumulation [39].

**Table 5:** COD, total nitrogen, and total phosphorus at the initial and final time of PHB production from HE using *Coelastrella* sp. KKU-P1 and *Acutodesmus* sp. KKU-P2 as the inoculum in the PBR.

Microalgae	COD (g/L)		COD consumption (%)	TN (mg/L)		Nitrogen consumption (%)	TP (mg/L)		Phosphorus consumption (%)
	Initial	Final		Initial	Final		Initial	Final	
KKU-P1	5.12 ± 0.04	2.42 ± 0.20	52.73^a^	99.30 ± 3.14	78.95 ± 2.54	20.49^a^	21.56 ± 0.58	6.67 ± 1.01	69.06^a^
KKU-P2	5.10 ± 0.03	3.14 ± 0.09	38.43^b^	94.74 ± 2.06	81.49 ± 1.67	13.99^b^	18.93 ± 0.40	8.31 ± 1.22	56.10^b^

Mean values are average of three replications ± standard deviation (n = 3). Values marked with the same letter are not significantly different (*p* < 0.05).

**Fig. 8:**
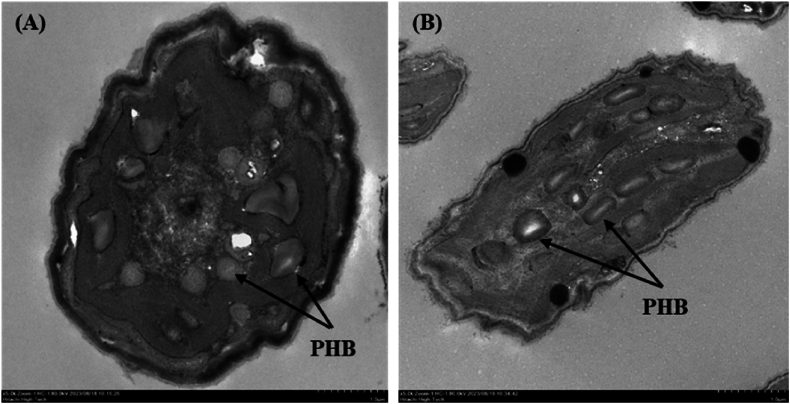
TEM images of intracellular PHB granules of *Coelastrella* sp. KKU-P1 (A) and *Acutodesmus* sp. KKU-P2 cultivated in NP-limited BBM under dark condition (B).

**Fig. 9:**
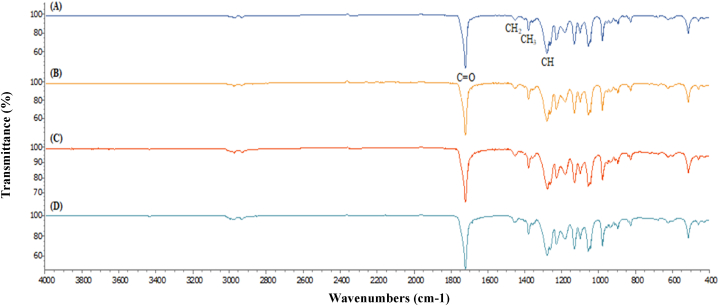
The FTIR spectrum of standard PHB (A), standard PHBV (B), extracted PHB from *Coelastrella* sp. KKU-P1 (C) and *Acutodesmus* sp. KKU-P2 (D).

The accumulation of PHB granule inside the microalgal cells was analyzed using TEM (Fig. 8). The TEM images revealed the presence of PHB granules in the cytoplasm of *Coelastrella* sp. KKU-P1 and *Acutodesmus* sp. KKU-P2 cells. The FTIR spectra of standard PHB, PHBV, extracted PHB from *Coelastrella* sp. KKU-P1, and *Acutodesmus* sp. KKU-P2 were shown in Fig. 9. The results found that the dominant peak was observed at the wave number 1721 cm^−1^, corresponding to carbonyl groups (C=O). Moreover, the peaks at the wave number 1453, 1379, and 1279 cm^−1^ corresponded to –CH_2_, –CH_3_, and –CH groups. These wave number were classified as the major functional group founded in the PHB and PHBV. Therefore, FTIR analyses confirmed that the extracted samples from KKU-P1 and KKU-P2 were PHB and PHBV. Results confirm that the KKU-P1 and KKU-P2 can utilize the HE for their growth and PHB production.

The biochemical composition, including proteins, carbohydrates, and lipids, of the biomass obtained at the initial and final PHB accumulation phases was examined, with the results depicted in Table 6. Initially, the protein content in KKU-P1 and KKU-P2 was 20.35 ± 1.70 % w/w and 23.76 ± 3.30 % w/w, respectively. This content increased to 29.37 ± 2.68 % w/w and 31.53 ± 3.20 % w/w by the end of the PHB accumulation phase. The increase in protein content correlated with the N concentration in the medium (Table 5). Consequently, KKU-P1 and KKU-P2 appeared to utilize the N source in the HE for protein accumulation, with N consumption rates of 20.49 % and 13.99 %, respectively (Table 5). The microalgal protein, rich in essential amino acids, is suitable as a feed supplement in livestock production [35]. Our results suggest that the increase in protein accumulation in KKU-P1 and KKU-P2 is associated with PHB synthesis. In contrast, the studies by Samadhiya, Ghosh [43] indicated that PHB production in *Ettlia texensis* was closely related to lipid synthesis. Nonetheless, PHB production in microalgal cells varies depending on the species and nutrient concentration.

**Table 6:** Protein, carbohydrate and lipid contents of *Coelastrella* sp. KKU-P1 and *Acutodesmus* sp. KKU-P2 at the initial and final time in the PHB accumulation stage.

Microalgae	Protein (% w/w)		Carbohydrate (% w/w)		Lipid (% w/w)	
	Initial	Final	Initial	Final	Initial	Final
KKU-P1	20.35 ± 1.70	29.37 ± 2.68^a^	14.77 ± 1.69	24.76 ± 1.83^a^	10.18 ± 1.36	13.21 ± 0.88^a^
KKU-P2	23.76 ± 3.30	31.53 ± 3.20^a^	22.53 ± 0.49	16.16 ± 2.88^b^	7.74 ± 1.11	4.77 ± 0.78^b^

Mean values are average of three replications ± standard deviation (n = 3). Values marked with the same letter are not significantly different (*p* < 0.05).

KKU-P1 and KKU-P2 can store carbohydrates during their growth, with initial carbohydrate contents of 14.77 % w/w and 22.35 % w/w, respectively. By the end of the PHB accumulation phase, the carbohydrate content of KKU-P1 had increased to 24.76 % w/w, whereas that of KKU-P2 had decreased to 16.16 % w/w. Under mixotrophic growth conditions, the carbohydrate accumulation of KKU-P2 significantly decreased, likely due to the availability of external organic sources for metabolic activities. The two primary pathways for carbon assimilation in microalgae, the EMP and the PPP, produce a substantial amount of energy, which can enhance cell growth and, consequently, reduce carbon storage [48].

Regarding lipid accumulation, the lipid content in KKU-P1 increased, whereas it decreased in KKU-P2 at the end of the PHB accumulation phase. This phenomenon mirrored that of carbohydrate metabolism. The lipid synthesis pathway is intertwined with carbohydrate metabolism. Under conditions of carbon excess, microalgae can produce various precursors such as malonyl-CoA and acetyl-CoA for lipid biosynthesis via glycolysis and the TCA cycle. N and P limitation lead to the accumulation of reactive oxygen species (ROS), which disrupt cell growth and energy assembly, and redirect carbon flux towards lipid accumulation. These limiting conditions have also been reported to favor the lipid biosynthesis pathway through the production of glutamine oxoglutarate aminotransferase (GOGAT). Furthermore, nutrient limitation is associated with triggering lipid accumulation, and due to shared metabolic precursors, lipid and PHB accumulation pathways often oppose each other. Certain studies have reported that *Chlorella* sp. [49]. and *Monodus subterraneus* [50] exhibit a close association between lipid accumulation and phosphorus concentration in the culture medium.

## 4 Conclusions

This study demonstrates the successful use of HE from biohydrogen production as a low-cost substrate for PHB production with two microalgal strains*, Coelastrella* sp. KKU-P1 and *Acutodesmus* sp. KKU-P2, in an upscaled PBR. Concentrations of HE greater than 10 % (v/v) were found to impede light penetration, resulting in reduced microalgal growth. Moreover, variations in nutrient composition and photoperiods led to differences in biomass production and PHB accumulation. The optimal condition for PHB accumulation with KKU-P1 and KKU-P2 was determined to be NP-limited BBM under dark conditions (without light). The results suggest that a mixotrophic approach under nutrient limitation conditions are favorable for PHB production with KKU-P1 and KKU-P2, highlighting the potential of HE as a low-cost feedstock for large-scale PHB production by microalgal strains.

## Funding

This project was supported by the 10.13039/501100004156Mahidol University Fundamental Fund 2023 (Grant No: FF-115/2566), Thailand. The project was also partially supported by the 10.13039/501100004704National Research Council of Thailand (NRCT) (Grant No. N42A670487).

## Data availability statement

Data will be made available upon request. The GenBank accession numbers for *Coelastrella* sp. KKU-P1 and *Acutodesmus* sp. KKU-P2 are MW581273 and MW555785, respectively.

## CRediT authorship contribution statement

**Kamolwan Thepsuthammarat:** Writing – original draft, Methodology, Investigation, Data curation. **Tsuyoshi Imai:** Visualization, Validation, Supervision. **Pensri Plangklang:** Visualization, Validation, Supervision, Resources. **Sureewan Sittijunda:** Writing – review & editing, Writing – original draft, Visualization, Validation, Supervision, Project administration, Conceptualization. **Alissara Reungsang:** Visualization, Validation, Supervision, Conceptualization.

## Declaration of competing interest

The authors declare that they have no known competing financial interests or personal relationships that could have appeared to influence the work reported in this paper.
